# Intervention Targets to Optimize Antibiotic Prescribing on Discharge from the Hospital to Nursing Homes

**DOI:** 10.1017/ash.2024.126

**Published:** 2024-09-16

**Authors:** Jon Furuno, Brie Noble, Dominic Chan, Thuan Nguyen, Andrea Hildebrand, Jessina McGregor, Emily Shephard, Sally Jolles, YoungYoon Ham, David Bearden, Caitlin McCracken, Seiko Izumi, Christopher Crnich

**Affiliations:** Oregon State University College of Pharmacy; Department of Quantitative Health Sciences, Mayo Clinic, Scottsdale, AZ, USA; Oregon Health & Science University; Oregon State University; Legacy Health; University of Wisconsin Madison Department of Medicine; University of Wisconsin

## Abstract

**Background:** Approximately half of antibiotics used in nursing home (NHs) are initiated in acute care hospitals prior to NH admission. Optimizing antibiotic prescribing on hospital discharge to these facilities presents an opportunity to improve NH antibiotic use. We aimed to identify intervention targets to optimize antibiotic use on discharge from the hospital to NHs. **Methods:** This was a multicenter, cross-sectional study across 9 acute care hospitals in Oregon, Wisconsin, and Washington. We selected a 20% random sample of adult (age >18) inpatients prescribed at least one antibiotic on discharge from the hospital to a NH between 2016-2018. We excluded patients discharged from the emergency department or an intensive care unit. Study data were electronically extracted from patients’ electronic health records and supplemented with manual chart review. Antibiotic optimization opportunities were determined by an infectious diseases (ID) physician or ID pharmacist and classified as definitely, possibly, or unlikely. Expert reviewers also recorded the type of optimization opportunity and the rationale for each determination. A gamma lasso algorithm was used to identify patient-level characteristics associated with definite optimization opportunity, which were then included in a logistic regression model. **Results:** There were 2761 antibiotic prescriptions among 2215 patients. Mean (standard deviation) age was 71.9 (14.3) years and 48.8% were male. Most discharges (83.1%) were prescribed one antibiotic, 15.2% were prescribed two antibiotics, and 1.8% were prescribed three antibiotics. The most frequently prescribed antibiotics were cephalexin (10.4%), vancomycin (9.8%), and amoxicillin clavulanate (8.4%). Among the 2761 antibiotic prescriptions, expert reviewers determined that 18.4% could definitely be optimized, 36.0% could possibly be optimized, and 45.3% unlikely could have been optimized. Among the 508 definite antibiotic optimization opportunities, 25.2% were to subtract the antibiotic, 56.3% were to change the antibiotic, 11.0% were to change the dose, 25.0% were to change the duration, 0.8% were to change the route, and 1.8% were to change the schedule. Patient-level characteristics found to be associated with definite antibiotic optimization opportunity included age over 80 years (odds ratio (OR)=1.44, 95% confidence interval (CI): [1.14, 1.82]), length of stay < 8 days (OR=1.40, 95% CI: [1.09, 1.81]), discharge with multiple antibiotic prescriptions (OR=1.92, 95% CI: [1.39, 2.63]), and discharge with prescription for oral vs intravenous (IV) antibiotics (OR=2.08, 95% CI: [1.49, 2.95]). **Conclusion:** We identified several patient and antibiotic characteristics which may serve as intervention targets to optimize antibiotic prescribing on discharge from the hospital to nursing homes.

